# Obesity: An independent protective factor for localized renal cell carcinoma in a systemic inflammation state

**DOI:** 10.1590/S1677-5538.IBJU.2019.0228

**Published:** 2020-10-30

**Authors:** Zhenhua Liu, Haifeng Wang, Yuke Chen, Jie Jin, Wei Yu

**Affiliations:** 1 Peking University First Hospital Peking University National Urological Cancer Center Beijing China Department of Urology, Peking University First Hospital and Institute of Urology, Peking University , National Urological Cancer Center , Beijing , China ;; 2 Department of Anesthesiology Peking University First Hospital Peking University Beijing China Department of Anesthesiology , Peking University First Hospital , Peking University , Beijing , China

**Keywords:** Obesity, Carcinoma, Inflammation

## Abstract

**Objectives:**

To explore the prognostic value of obesity (measured by BMI) on RCC in a systemic inflammation state.

**Patients and Methods:**

Clinicopathological and hematological data of 540 surgically treated Chinese localized RCC patients between 2005 and 2010 were retrospectively collected. Found by receiver operating characteristic (ROC) curve for cancer-specific survival (CSS), the optimal cutoff values of neutrophil-lymphocyte ratio (NLR, an indicator of systemic inflammation state) and BMI were 2.12 and 23.32, respectively. Survival curves were drawn using Kaplan-Meier method. Univariate and multivariate Cox regression analyses were used to evaluate the prognostic value of BMI in localized RCC patients with different NLR.

**Results:**

Overall, 36 patients died with a median follow-up of 70 months. Median overall survival (OS) was 66 months and the 5-year OS rate was 92.7%. In the multivariate analysis of total patients, higher BMI was an independent protective factor for CSS in total patients (p=0.048). While in systemic inflammation subgroup (high NLR subgroup) patients, higher BMI (obesity) turned out to be an independent protective factor for both CSS (p=0.025) and RFS (p=0.048).

**Conclusion:**

In localized RCC patients, obesity was an independent protective factor for CSS and RFS in a systemic inflammation state.

## INTRODUCTION

Renal cell carcinoma (RCC) is the most common malignancy of kidney, accounting for 2%-3% of all adult malignancies ( 1 ). 20%-40% of localized RCC patients still suffered from cancer recurrence or metastasis even after surgery treatment, despite the significant improvement of RCC therapy ( 2 ). Thus, it is of importance to find effective prognostic factors to facilitate progress in treatment strategy.

Obesity is a widely accepted risk factor for the onset of RCC ( 3 , 4 ). As an indicator of obesity, body mass index (BMI) was widely studied for its effect on the prognosis of RCC. Nevertheless, although obesity increases the incidence of RCC, several previous studies have shown that RCC patients with higher BMI at diagnosis might have better survival outcomes than those with normal or lower BMI levels ( 5 - 7 ). However, some investigators fail to confirm the existence of such association ( 8 , 9 ). Although increasing evidence supports that higher BMI is a favorable prognostic factor of RCC, this topic has not been thoroughly explored.

Increased neutrophil-lymphocyte ratio (NLR) is significantly associated with insulin resistance (IR), which is considered the common cause of impaired glucose tolerance, diabetes, dyslipidemia, hypertensive diseases and obesity ( 10 ). And accumulating evidence suggests that high NLR might be an adverse prognostic factor in metastatic RCC patients treated with interferon, interleukin-2 or sunitinib ( 11 - 13 ). However, studies regarding the prognostic value of NLR in non-metastatic RCC remain sparse.

NLR is an easily accessible index and high NLR has been proposed as an indicator of systemic inflammatory response, which is independently associated with clinical outcomes of various cancers ( 14 ). A systemic inflammatory state may be established long before metastases become clinically evident ( 15 ). Thus, it is of importance to study the prognostic effect of BMI under systemic inflammation state.

## MATERIALS AND METHODS

### Study population

Our retrospective study included 540 patients with localized renal cell carcinoma who underwent curative surgeries in Peking University First Hospital between 2005 and 2010. Patient’s collection was based on the following inclusion criteria: ( 1 ) patients who were pathologically diagnosed with localized RCC (pT1-2N0M0, p: pathological grading) after surgery, ( 2 ) patients with complete information about BMI and NLR, ( 3 ) patients who had at least one effective follow-up. Patients were excluded if they had any of the following condition: ( 1 ) patients with previously diagnosed cancers or autoimmune diseases, ( 2 ) patients with incomplete clinical or pathological data, ( 3 ) patients who underwent previous chemotherapy and/or radiation therapy. This study was approved by the institutional ethics committee of Peking University First Hospital. As a retrospective analysis of routine data, a waiver of written informed consent was granted from the ethics committee. Patient records/information was anonymized and de-identified prior to analysis.

### Clinical and pathological data collection

Clinicopathological and hematological data including gender (female or male), age (years old), height (m), weight (kg), cancer related symptoms (absent or present), histological subtype (clear cell or non-clear cell), Fuhrman nuclear grade (1-2 or 3-4), tumor necrosis (no or yes), tumor laterality (left or right), tumor size (≤7cm or >7cm, equals to T1 or T2 in TNM staging system), surgical procedures (partial or radical), neutrophil counts and lymphocyte counts were collected from medical records in the Department of Urology, Peking University First Hospital. Pathological TNM stage for each RCC was determined according to the AJCC 2002 TNM staging system. Patients were closely followed up after discharge with regular post-operative tests. BMI (kg/m ^2^ ) was calculated based on the measurements of height and weight at diagnosis. NLR was calculated as preoperative neutrophil counts divided by lymphocyte counts. The optimal cut-off value of BMI (23.32) and NLR (2.12) were determined according to the receiver operating characteristic (ROC) curves (shown in supplementary Figures S.1 and S.2 of cancer-specific survival (CSS). According to the Asian and Chinese standard of obesity, the normal ceiling of BMI is 23-24 ( 16 , 17 ), and our cut-off value (23.32) falls in this range.

### Indicators of prognosis

Overall survival (OS), cancer-specific survival (CSS) and recurrence-free survival (RFS) were used as indicators of prognosis of the localized RCC patients in the study. OS, CSS, RFS were the intervals between the date of surgery treatment and ( 1 ) the date of death or last follow-up, ( 2 ) cancer-related death or last follow-up, ( 3 ) radiologic or histological confirmation of cancer recurrence or last follow-up.

### Statistical analysis

**Figure 1:**
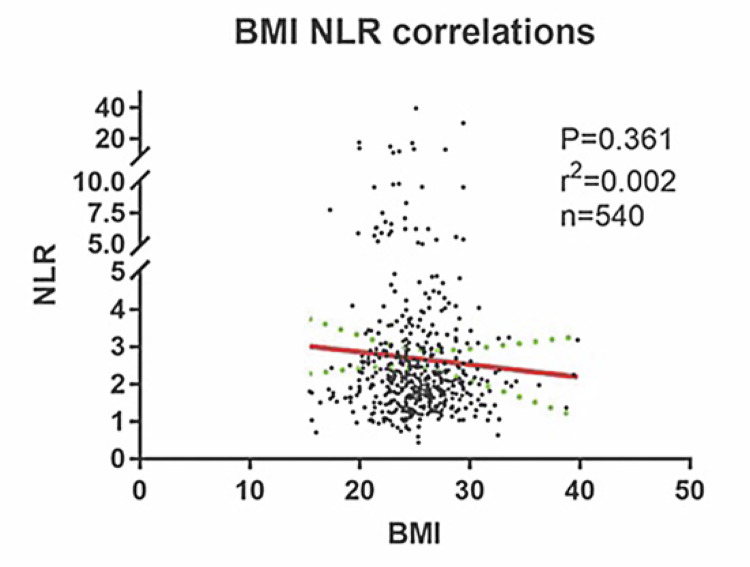
Correlation analysis between BMI and NLR. No correlation could be found between BMI and NLR (p >0.05). Abbreviation: **BMI** = body mass index, **NLR** = neutrophil-lymphocyte ratio

**Figure 2:**
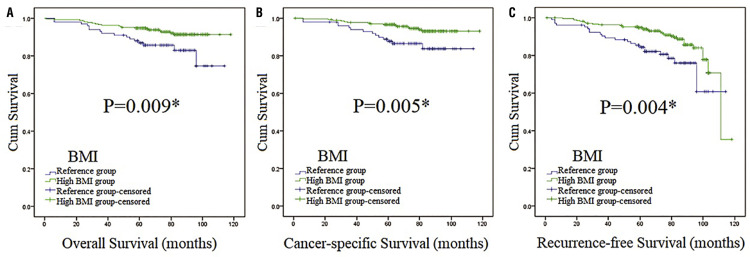
Survival curves stratified by BMI at the level of 23.32 in total patients (Kaplan-Meier method). Survival curves of OS (Figure 2A), CSS (Figure 2B) and RFS (Figure 2C) in total patients. *:p <0.05; Abbreviation: BMI=body mass index

The clinicopathological characteristics between groups with different BMI were compared using chi-square test. Kaplan-Meier survival curves were compared by using the log-rank test. BMI and other variables with P <0.1 in univariate analysis were included in the multivariate Cox proportional hazards regression model, and P <0.05 (labeled with ‘*’) was regarded as statistically significant. Also, by using Cox proportional hazards regression models, we obtained the hazard ratios (HRs) and 95% confidence intervals (CIs) from the survival time. Data were analyzed using IBM SPSS statistics software (version 22.0, SPSS Inc., Chicago, IL, USA) for Microsoft Windows. Pictures were drawn by GraphPad Prism (version7, Graphpad software, Inc., La Jolla, CA, USA, Figure 1 ) and IBM SPSS statistics software ( Figure-2 ).

## RESULTS

### Cohort characteristics

In total, 400 men and 140 women with localized RCC were included in the study with a mean age of 54±13.4 years old. 48.3% (261/540) of the tumors were located on the left side. Only 53 (9.8%) patients manifested cancer related symptoms (backache, hematuria and/or abdominal mass). Most patients (88.9%, 480/540) suffered from clear cell carcinoma. Patients whose tumor size was bigger than 7cm accounted for less than 10%. Using 23.32 as the cutoff value of BMI, 145 (26.9%) and 395 (73.1%) patients were respectively stratified into the reference group (low BMI group BMI <23.32) and high BMI group (BMI ≥23.32). Differences in gender were found between reference group and high BMI group p=0.006). No differences were found between reference group and high BMI group in terms of age, tumor laterality, cancer related symptoms presence, histology, tumor size, Fuhrman nuclear grade, tumor necrosis or NLR. 283 and 257 patients were respectively categorized into low and high NLR group, according to the cutoff value of NLR at 2.12 ( Table-1 ). Correlation analysis found no correlation between NLR and BMI ( Figure-1 ).

**Table 1:** Distribution of clinicopathological factors stratified by preoperative BMI.

Characteristics	All patients	BMI<23.32	BMI≥23.32	P value

	N=540 (N%)	N=145 (N%)	N=395 (N%)	
**Age**				**0.184**
<60	352 (65.2)	88 (60.7)	264 (66.8)	
≥60	188 (34.8)	57 (39.3)	131 (33.2)	
**Gender**				0.006*
Male	400 (74.1)	95 (65.5)	305 (77.2)	
Female	140 (25.9)	50 (34.5)	90 (22.8)	
**Tumor laterality**				**0.428**
Left	261 (48.3)	66 (45.5)	195 (49.4)	
Right	279 (51.7)	79 (54.5)	200 (50.6)	
**Cancer related symptoms**				**0.219**
Absent	487 (90.2)	127 (87.6)	360 (91.1)	
Present	53 (9.8)	18 (12.4)	35 (8.9)	
**Surgical procedures**				
Partial resection	157 (29.1)	40 (27.6)	118 (29.9)	**0.605**
Radical resection	383 (70.9)	105 (72.4)	277 (70.1)	
**Histology**				**0.131**
Clear cell	480 (88.9)	124 (85.5)	356 (90.1)	
Non-clear cell	60 (11.1)	21 (14.5)	39 (9.9)	
**Tumor size**				**0.066**
≤7 (T1N0M0)	496 (91.9)	128 (88.3)	368 (93.2)	
>7 (T2N0M0)	44 (8.1)	17 (11.7)	27 (6.8)	
**Fuhrman Grade**				**0.085**
1-2	479 (88.7)	123 (84.8)	356 (90.1)	
3-4	61 (11.3)	22 (15.2)	39 (9.9)	
**Tumor necrosis**				**0.163**
Absent	462 (85.6)	119 (82.1)	343 (86.8)	
Present	78 (14.4)	26 (17.9)	52 (13.2)	
**NLR**				**0.244**
≤2.12	283 (52.4)	70 (48.3)	213 (53.9)	
>2.12	257 (47.6)	75 (51.7)	182 (46.1)	

*:p<0.05; Abbreviations: **BMI** =body mass index; **NLR** =neutrophil-lymphocyte ratio.

The data of histology, tumor size, Fuhrman Grade and tumor necrosis were obtained from pathological findings of surgical specimens.

### Survival analysis on BMI and NLR

**Figure 3:**
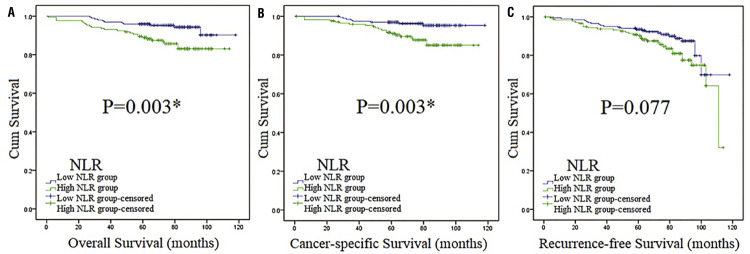
Survival curves stratified by NLR at the level of 2.12 in total patients (Kaplan-Meier method). Survival curves of OS (Figure 3A), CSS (Figure 3B) and RFS (Figure 3C) in total patients. Abbreviation: **NLR** = neutrophil-lymphocyte ratio. *:p <0.05

**Figure 4:**
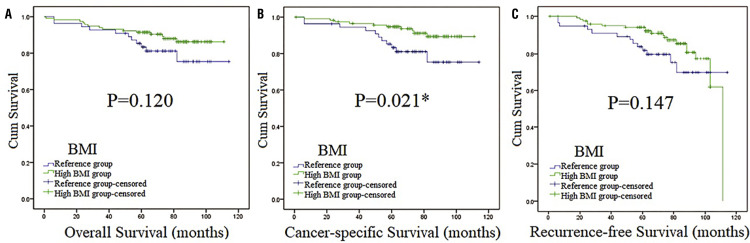
Survival curves stratified by BMI at the level of 23.32 in high NLR patients (Kaplan-Meier method). Survival curves of OS (Figure 4A), CSS (Figure 4B) and RFS ( Figure 4C ) in high NLR patients. Abbreviations: **BMI** =body mass index; **NLR** =neutrophil-lymphocyte ratio. *:p <0.05

**Figure 5:**
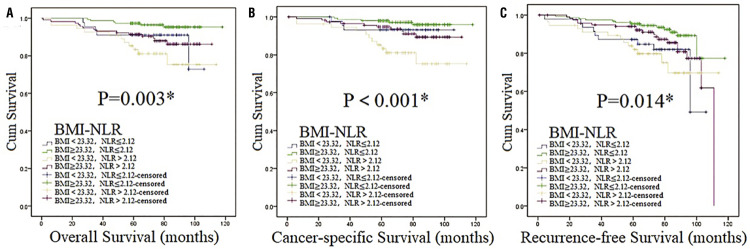
Survival curves stratified by BMI-NLR at the level of 23.32 (BMI) and 2.12 (NLR) in total patients (Kaplan-Meier method). Survival curves of OS ( Figure 5A ), CSS ( Figure 5B ) and RFS ( Figure 5C ) in total patients. Abbreviations: **BMI** =body mass index; **NLR** =neutrophil-lymphocyte ratio. *:p <0.05

We used OS, CSS and RFS as the indicators of prognosis to estimate the association of BMI and the clinical outcomes of patients with localized RCC. The median follow-up was 70 months (in the range of 1-118). Overall, 36 patients died, with 16 and 20 in the reference group and high BMI group, respectively. Median OS was 66 months (in the range of 1-118) and the 5-year OS rate was 92.7%. Kaplan-Meier (K-M) curves indicated that differences were found between reference group and high BMI group in OS, CSS and RFS, establishing that BMI was associated with OS, CSS and RFS in our study ( Figure-2 ). Also, high BMI group had higher survival curves of OS, CSS and RFS than the reference group, which indicated that high BMI group might have better OS, CSS and RFS. On the other hand, patients with high NLR had worse OS and CSS than low NLR according to survival curves ( Figure-3 ), which indicated they had worse OS and CSS. Then, subgroup analysis was performed by stratifying subjects by NLR at the level of 2.12. As we could see in Figure-S.3 , BMI was associated with RFS (p=0.010). While in high NLR group patients ( Figure 4 ), association was found between BMI and CSS (p=0.021). In Figure-5 , K-M curves were drawn using a combination of BMI and NLR, of which high BMI-low NLR subgroup had best survival outcomes, while low BMI-high NLR subgroup was the worst.

### Univariate and multivariate analysis

Univariate Cox regression analyses of factors for OS, CSS and RFS were shown in Tables 2-4 . [Table t3] [Table t4] In univariate analysis of total patients, larger tumor size (>7 vs. ≤7cm) and lower BMI (<23.32 vs. ≥23.32) were associated with poorer OS, CSS and RFS (all p <0.05). Older age (≥60 vs. <60 years) and higher NLR (>2.12 vs. ≤2.12) were correlated with lower OS (p <0.001, p=0.005, respectively) and CSS (p=0.003, p=0.005, respectively). Presence of cancer related symptoms was associated with worse CSS (p=0.012) and RFS (p=0.001). Radical nephrectomy (radical vs. partial) and higher Fuhrman grade (3-4 vs. 1-2) were correlated with poorer OS (p=0.047, p=0.004, respectively) and RFS (p <0.030, p <0.001, respectively). Gender, tumor laterality, histology and tumor necrosis were not associated with OS, CSS or RFS (all p >0.05).

**Table 2:** Univariate and multivariate analysis of OS in total patients, low NLR and high NLR subgroups.

Characteristics	Total						Low NLR						High NLR (systemic inflammation state)				

	Univariate analysis			Multivariate analysis			Univariate analysis			Multivariate analysis			Univariate analysis			Multivariate analysis	

	HR (95%CI)	P		HR (95%CI)	P		HR (95%CI)	P		HR (95%CI)	P		HR (95%CI)	P		HR (95%CI)	P
**Gender (female vs. male)**	0.574 (0.252-1.311)	0.188					0.790 (0.210-2.978)	0.728					0.517 (0.177-1.507)	0.227			
**Age (<60 vs. ≥60)**	3.734 (1.867-7.468)	<0.001*		4.010 (1.987-8.091)	<0.001		9.482 (2.048-43.911)	0.004*		9.040 (1.948-41.949)	0.005*		2.471 (1.110-5.501)	0.027*		3.348 (1.424-7.873)	0.006*
**Tumor laterality (left vs. right)**	1.417 (0.730-2.749)	0.303					1.330 (0.406-4.361)	0.638					1.379 (0.619-3.072)	0.431			
**Cancer related symptoms (absent vs. present)**	2.322 (0.965-5.589)	0.06		3.073 (1.241-7.614)	0.015*		2.116 (0.455-9.828)	0.339					2.736 (0.938-7.978)	0.065		3.872 (1.263-11.869)	0.018*
**Surgical procedures (partial vs. radical)**	2.603 (1.012-6.699)	0.047*					5.677 (0.726-44.349)	0.098					1.465 (0.503-4.271)	0.484			
**Tumor size (≤7 vs. >7)**	2.778 (1.156-6.677)	0.022*		2.483 (1.022-6.030)	0.045*		2.358 (0.302-18.444)	0.414					2.290 (0.859-6.108)	0.098		2.856 (1.047-7.793)	0.040*
**Histology (clear cell vs. non-clear cell)**	1.085 (0.384-3.070)	0.877					0.675 (0.146-3.131)	0.616					1.424 (0.336-6.040)	0.632			
**Tumor necrosis (no vs yes)**	1.563 (0.684-3.573)	0.29					3.346 (0.879-12.731)	0.077					0.935 (0.321-2.726)	0.902			
**Fuhrman grade (1-2 vs. 3-4)**	3.051 (1.433-6.498)	0.004*					4.377 (1.153-16.615)	0.030*		3.887 (1.001-15.098)	0.0499*		2.200 (0.877-5.520)	0.093			
**NLR (≤2.12 vs. >2.12)**	2.762 (1.359-5.614)	0.005*		2.761 (1.340-5.690)	0.006*												
**BMI (<23.32 vs.≥ 23.32)**	0.428 (0.222-0.828)	0.012*					0.334 (0.102-1.097)	0.071					0.540 (0.244-1.191)	0.127			

* *:p* < 0.05; Abbreviations: **BMI** =body mass index; **NLR** =neutrophil-lymphocyte ratio; **HR** =hazard ratio; **CI** =confidence interval; **OS** =overall survival; /=not significant; **Blank space** =not done.

**Table 3 t3:** Univariate and multivariate analysis of CSS in total patients, low NLR and high NLR subgroups.

Characteristics	Total				Low NLR				High NLR (systemic inflammation state)			

	Univariate analysis		Multivariate analysis		Univariate analysis		Multivariate analysis		Univariate analysis		Multivariate analysis	

	HR (95%CI)	P	HR (95%CI)	P	HR (95%CI)	P	HR (95%CI)	P	HR (95%CI)	P	HR (95%CI)	P
**Gender (female vs. male)**	0.752 (0.321-1.761)	0.511			0.711 (0.143-3.522)	0.676			0.839 (0.307-2.292)	0.732		
**Age (<60 vs. ≥ 60)**	3.084 (1.456-6.530)	0.003*	3.024 (1.339-6.827)	0.003*	6.326 (1.276-31.365)	0.024*	6.326 (1.276-31.365)	0.024*	2.198 (0.926-5.218)	0.074	2.991 (1.165-7.682)	0.023*
**Tumor laterality (left vs. right)**	1.251 (0.602-2.601)	0.549			1.103 (0.276-4.414)	0.89			1.236 (0.521-2.936)	0.631		
**Cancer related symptoms (absent vs. present)**	3.146 (1.280-6.847)	0.012*	4.597 (1.746-12.101)	0.003*	3.328 (0.669-16.547)	0.142			3.415 (1.148-10.164)	0.027*	5.285 (1.624-17.200)	0.006*
**Surgical procedures (partial vs. radical)**	2.599 (0.904-7.469)	0.076			3.851 (0.474-31.308)	0.207			1.689 (0.497-5.736)	0.401		
**Tumor size (≤7 vs. >7)**	3.641 (1.481-8.949)	0.005*	2.889 (1.160-7.195)	0.023*	3.363 (0.413-27.387)	0.257			2.901 (1.061-7.930)	0.038*	3.968 (1.420-11.086)	0.009*
**Histology (clear cell vs. non-clear cell)**	1.168 (0.353-3.859)	0.799			0.435 (0.088-2.156)	0.308			2.484 (0.333-18.514)	0.375		
**Tumor necrosis (no vs yes)**	1.032 (0.359-2.969)	0.953			2.802 (0.563-13.933)	0.208			0.521 (0.121-2.237)	0.38		
**Fuhrman grade (1-2 vs. 3-4)**	2.410 (0.980-5.928)	0.055			4.076 (0.821-20.242)	0.086			1.622 (0.544-4.831)	0.385		
**NLR (≤2.12 vs. >2.12)**	3.212 (1.422-7.254)	0.005*	3.360 (1.453-7.769)	0.005*								
**BMI (<23.32 vs.≥ 23.32)**	0.363 (0.175-0.753)	0.006*	0.474 (0.226-0.994)	0.048*	0.461 (0.110-1.933)	0.29			0.378 (0.160-0.893)	0.027*	0.367 (0.153-0.879)	0.025*

*:p<0.05; Abbreviations: **BMI** =body mass index; **NLR** =neutrophil-lymphocyte ratio; **HR** =hazard ratio; **CI** =confidence interval; **CSS** =cancer specific survival; /=not significant; **Blank space** =not done.

**Table 4 t4:** Univariate and multivariate analysis of RFS in total patients, low NLR and high NLR subgroups.

Characteristics	Total				Low NLR					High NLR (systemic inflammation state)				

	Univariate analysis		Multivariate analysis		Univariate analysis			Multivariate analysis		Univariate analysis			Multivariate analysis	

	HR (95%CI)	P	HR (95%CI)	P	HR (95%CI)		P	HR (95%CI)	P	HR (95%CI)		P	HR (95%CI)	P
**Gender (female vs. male)**	0.707 (0.380-1.315)	0.273				0.836 (0.346-2.022)	0.692				0.613 (0.251-1.469)	0.282		
**Age (<60 vs. ≥ 60)**	1.494 (0.876-2.548)	0.14				2.277 (1.019-5.088)	0.045				1.027 (0.498-2.117)	0.943		
**Tumor laterality (left vs. right)**	0.922 (0.545-1.558)	0.761				1.065 (0.478-2.371)	0.878				0.840 (0.419-1.685)	0.624		
**Cancer related symptoms (absent vs. present)**	3.065 (1.610-5.833)	0.001*	2.501 (1.294-4.836)	0.006*		2.061 (0.701-6.055)	0.189				4.429 (1.968-9.967)	<0.001*t	5.671 (2.393-13.440)	<0.001*
**Surgical procedures (partial vs. radical)**	2.210 (1.080-4.522)	0.030*				1.370 (0.567-3.312)	0.484				4.398 (1.046-18.500)	0.043*	4.151 (0.929-18.545)	0.062
**Tumor size (≤7 vs. >7)**	4.823 (2.656-8.757)	<0.001*	3.837 (2.048-7.187)	<0.001*		3.049 (0.900-10.332)	0.073				5.737 (2.757-11.937)	<0.001*	4.574 (2.125-9.848)	<0.001*
**Histology (clear cell vs. non-clear cell)**	1.769 (0.639-4.897)	0.273				1.134 (0.336-3.825)	0.84				3.706 (0.505-27.184)	0.198		
**Tumor necrosis (no vs yes)**	1.476 (0.741-2.941)	0.268				2.602 (0.955-7.094)	0.062				0.911 (0.346-2.402)	0.851		
**Fuhrman grade (1-2 vs. 3-4)**	2.957 (1.626-5.378)	<0.001*	1.990 (1.051-3.768)	0.035*		4.923 (1.996-12.144)	0.001*	4.923 (1.996-12.144)	0.001*		1.885 (0.833-4.266)	0.128		
**NLR (≤2.12 vs. >2.12)**	1.604 (0.944-2.726)	0.081												
**BMI (<23.32 vs.≥ 23.32)**	0.467 (0.274-0.797)	0.005*				0.358 (0.157-0.815)	0.014*				0.595 (0.292-1.211)	0.152	0.477 (0.229-0.994)	0.048*

*:p<0.05; Abbreviations: **BMI** =body mass index; **NLR** =neutrophil-lymphocyte ratio; **HR** =hazard ratio; **CI** =confidence interval; **RFS** =recurrence-free survival; /=not significant; **Blank space** =not done.

Subgroup univariate analysis revealed that in low NLR group patients, older age remained its association with lower OS (p=0.004) and CSS (p=0.024). Higher Fuhrman nuclear grade had correlation with poorer OS (p=0.030) and RFS (p=0.001). Larger BMI value was associated with better RFS (p=0.014). While in high NLR group (systemic inflammation state) patients, older age was associated with worse OS (p=0.027). Manifestation of cancer related symptoms and larger tumor size were correlated with worse CSS (p=0.027, p=0.038, respectively) and RFS (both p <0.001), but not OS. Radical nephrectomy (radical vs. partial) was associated with poorer RFS (p=0.043). Larger BMI value was correlated with better CSS (p=0.027).

Outcomes of multivariate Cox regression analysis of OS, CSS and RFS are listed in Tables 2-4 [Table t3] [Table t4] . In the multivariate analysis of total patients, manifestation of cancer related symptoms and larger tumor size were independent risk factors for OS (p=0.015; p=0.045, respectively), CSS (p=0.003; p=0.023, respectively) and RFS (p=0.006; p <0.001, respectively). Older age and higher NLR had independent adverse effects on OS (p <0.001; p =0.006, respectively) and CSS (p=0.003; p=0.005, respectively). While higher BMI was only an independent protective factor for CSS (HR=0.474, 95%CI: 0.226-0.994, p=0.048).

Furthermore, subgroup multivariate analysis turned out that in low NLR subgroup, older age was an independent risk factor for OS (p=0.005) and CSS (p=0.024), higher Fuhrman grade was an independent adverse predictor for OS (p=0.0499) and RFS (p=0.001). While in high NLR group (systemic inflammation state) patients, presence of cancer related symptoms and larger tumor size became independent risk factors for OS, CSS and RFS (cancer related symptoms presence: p=0.018, p=0.006, p <0.001, respectively; tumor size: p=0.040, p=0.009, p <0.001, respectively). Also, older age was an independent adverse predictor for OS (p=0.006) and CSS (p=0.023). Interestingly, higher BMI turned out to be an independent protective factor for CSS (HR=0.367, 95%CI: 0.153-0.879, p=0.025) and RFS (HR=0.477, 95%CI: 0.229-0.994, p=0.048) in high NLR group (systemic inflammation state) patients.

## DISCUSSION

This study evaluated the prognostic value of BMI both in total patients and in systemic inflammation state patients. To the best of our knowledge, this is the first study to explore the prognostic value of obesity for localized renal cell carcinoma in a systemic inflammation state.

### BMI and RCC prognosis

Obesity has emerged as a significant adverse predictor for RCC in previous studies. People with an increased BMI have two to three folds increased risk for developing RCC ( 18 ). The hypothetical explanations for the increased risk included the alteration of the insulin-like growth factor system, lipid peroxidation, high levels of estrogen, hypertension and the malfunction of immune system. However, there seems to be a paradox: obesity increases the risk of RCC but in the meantime, it is associated with improved tumor prognosis. In our study, obesity was an independent favorable prognostic factor for CSS in total patients. The results were in line with some Asian studies. Jeon et al. found overweight and obese Korean patients with RCC had more favorable prognosis than those with a normal BMI ( 19 ). Similar researches by Awakura et al. also reported that a BMI of 23kg/m ^2^ or more favorably affected the prognosis of Japanese RCC subjects, although BMI did not differ significantly with respect to stage or grade ( 20 ).

Similar results have also been obtained in some studies about western RCC patients. Yu et al. suggested that prognosis was no worse and may even be better among obese patients with RCC ( 21 ). In a study of 400 patients with non-metastatic, node-negative RCC conducted by Kamat et al., overweight and obese patients had a more favorable prognosis than patients with a normal BMI ( 7 ). In a study composed of 970 clear cell RCC patients, Parker et al. reported that high BMI was associated with negative lymph nodes and the absence of metastases ( 5 ).

Some hypotheses had been proposed to explain the contradiction. Patients with higher BMI might have better nutritional status and potential survival advantage ( 22 ). RCC developed in the obese might represent biologically distinct and less aggressive tumors versus those with normal weight ( 6 , 23 ). Furthermore, patients with higher BMI were more likely to have contact with their physicians and have increased possibilities of early cancer detection ( 5 ).

The discovery of a new paradox inside the abovementioned paradox made the issue even more complicated. Bagheri et al. discovered through 8 studies of 8699 survivals that while CSS increased with BMI, when BMI is higher than 25, OS surprisingly decreased with BMI. Different causes of mortality had different directions after BMI reached a certain level, creating a ‘paradox within a paradox’ ( 24 ).

If we are to truly understand the role that BMI plays in RCC and other cancer patient, efforts are still needed to explicitly illustrate the issue in the future.

### Systemic inflammation state and RCC prognosis

NLR has been recognized as the representative hematological index of systemic inflammation ( 14 ). However, studies about the prognostic value of the pretreatment NLR in non-metastatic RCC are sparse and with conflicting findings. Ohno et al. found that an increased NLR was an independent risk predictor for relapse-free survival in a small cohort of 192 RCC patients from Japan ( 25 ). Interestingly, Pichler et al. demonstrated that an increased NLR was an independent negative predictor for OS ( 26 ). Variance in study designs and sample sizes might bring about different outcomes. Considering the uncertainty of NLR’s role in RCC patients, Hu performed a meta-analysis to assess the prognostic significance of high NLR for OS and RFS/PFS (progress-free survival), and found elevated NLR predicted poorer OS and RFS/PFS in patients with RCC ( 27 ).

As an index of systemic inflammation, high NLR might represent an inflammatory microenvironment which can increase mutation rates, in addition to enhancing the proliferation of mutated cells ( 28 ). High NLR is associated with high infiltration of tumor-associated macrophages (TAMs) which are identified to mediate refractoriness to anti-vascular endothelial growth factor (VEGF) treatment ( 29 ). Thus, that elevated NLR is related to poorer prognostic outcome of patients with RCC sounds reasonable. In Hu’s meta-analysis, pooled analysis of studies showed NLR played a far more superior prognostic role with a cutoff value no more than 3 compared to higher than 3 ( 27 ). So, choosing 2.12 as the cutoff value seems applicable (In our study, the optimal cutoff value of NLR was 2.12 for CSS, calculated by ROC curve).

In this research, NLR was associated with OS and CSS in total patients in univariate and multivariate analyses, which showed that high NLR (systemic inflammation state) was independently associated with poor OS and CSS. Therefore, our findings are in support of the recognition that systemic inflammation state has a correlation with poor outcomes in RCC patients.

One aspect of importance is to distinguish systemic inflammation from chronic inflammation common in all obese people. Chronic inflammation happens because adipose tissue secretes pro-inflammatory cytokines, leading to a state of chronic low-grade inflammation associated with obesity, such that obese persons often experience higher concentrations of inflammatory biomarkers than their normal-weight counterparts ( 30 ). Systemic inflammation state in our research, however, is defined by high NLR (>2.12), which is a much more severe condition than chronic inflammation above.

### Prognostic value of BMI in a systemic inflammation state

As can be inferred from discussion above, the prognostic value of BMI was greatly enhanced in a systemic inflammation state. Systemic inflammation state makes possible a favorable environment for cancer cells: infiltration and metastasis are relatively easier ( 31 ). Therefore, cancer patients will become particularly sensitive in this condition and react more fiercely to changes in a lot of conditions. BMI is one example. As discussed above, patients with higher BMI tend to be in better nutritional conditions and vice versa; the influence of BMI on prognosis is exponentially magnified in a systemic inflammation condition. The results of our study validated this hypothesis ( Figure-5 ). In subgroup analyses of our study, BMI was an independent favorable factor for CSS and RFS in high NLR (systemic inflammation state) patients rather than in low NLR patients, which indicated that the prognostic value of BMI was increased in systemic inflammatory status.

Another possible explanation includes the change in the effects of proinflammatory cytokines in subjects with high BMI. This is the case because obese patients are known to be associated with a state of chronic inflammation ( 23 ). Cytokines including C-reactive protein (CRP), tumor necrosis factor (TNF), IL6 and IL18, among others already increased greatly these patients ( 32 ). When this is the case, the effect of systemic inflammation (marked by high NLR) is attenuated since the body is already accustomed to abundance in inflammation cytokines. Therefore, the difference between survival outcomes between high and low BMI patients is further magnified, making it an especially sensitive independent predictor in a high NLR environment.

There are still some limitations in our study. As a retrospective study, selection bias was inevitable for making certain inclusion criteria. Another limitation was the relatively small size of samples from one single medical center. Our findings should be interpreted with caution until they are validated in a large multi-institutional pooled analysis.

If our finding that higher NLR increases the prognostic value of BMI is confirmed, BMI and NLR might need to be incorporated into the equation when protocols for therapy in RCC patients are planned.

## CONCLUSIONS

In localized RCC patients, BMI was an independent favorable factor for CSS. In subgroup analyses, BMI was an independent protective factor for CSS in high NLR patients rather than in low NLR patients, which indicated that the prognostic value of BMI was increased in systemic inflammatory status.

## Figures and Tables

**Figure:**
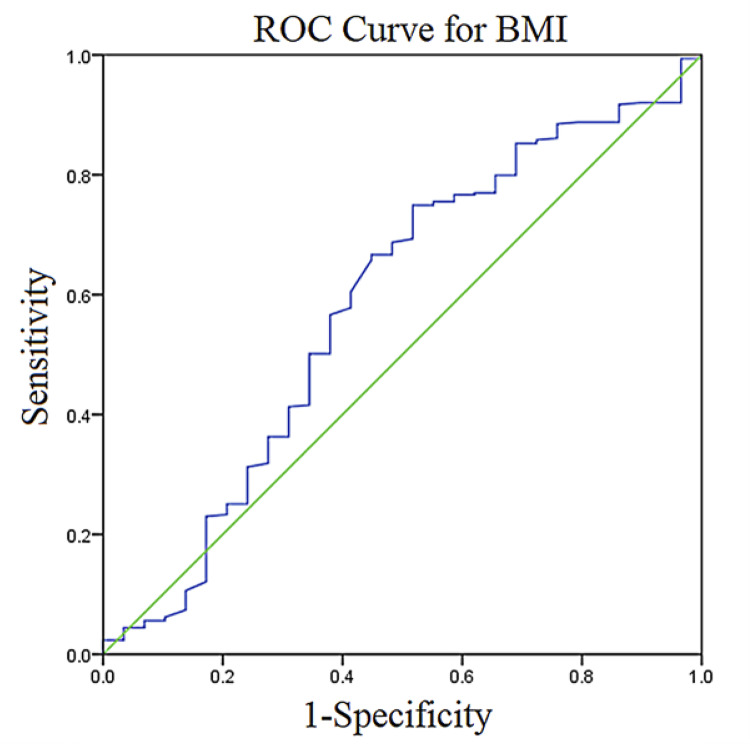
Figure-S.1 - Receiver operating characteristic (ROC) curve for BMI. Abbreviations: **BMI** =body mass index.

**Figure:**
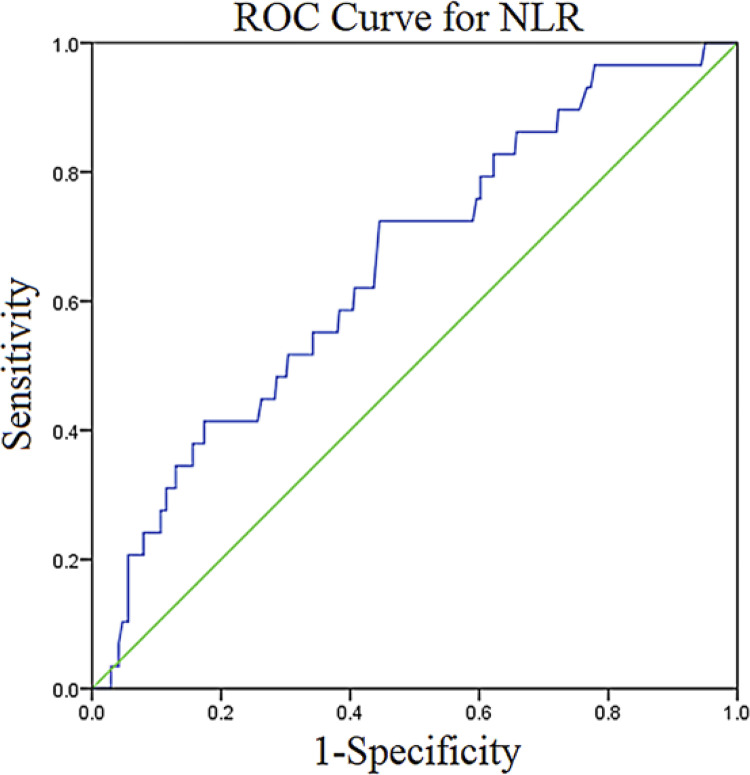
Figure-S.2- Receiver operating characteristic (ROC) curve for NLR. Abbreviations: **NLR=** neutrophil-lymphocyte ratio.

**Figure:**
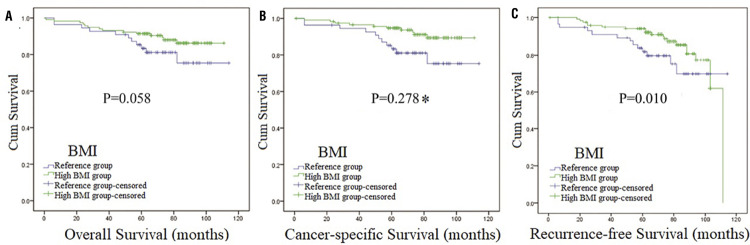
Figure-S.3- Survival curves stratified by BMI at the level of 23.32 in low NLR patients (Kaplan-Meier method). Survival curves of OS (Figure S.3A), CSS (Figure S.3B) and RFS (Figure S.3C) in low NLR patients. Abbreviations: **BMI** =body mass index; **NLR** =neutrophil-lymphocyte ratio. *:p <0.0
